# Diffuse large B‐cell lymphoma with cardiac invasion diagnosed using transesophageal ultrasound‐guided bronchoscopic aspiration

**DOI:** 10.1002/rcr2.1022

**Published:** 2022-08-15

**Authors:** Daiki Nagayama, Toshiyuki Sumi, Yoshiko Keira, Yusuke Tanaka, Haruhiko Michimata, Yuta Koshino, Hiroki Watanabe, Yuichi Yamada, Hirofumi Chiba

**Affiliations:** ^1^ Department of Pulmonary Medicine Hakodate Goryoukaku Hospital Hokkaido Japan; ^2^ Department of Respiratory Medicine and Allergology Sapporo Medical University School of Medicine Sapporo Japan; ^3^ Department of Surgical Pathology Hakodate Goryoukaku Hospital Hokkaido Japan

**Keywords:** cardiac invasion, DLBCL, EUS‐B‐FNA, mediastinal tumour, transesophageal approach

## Abstract

Transesophageal ultrasound‐guided bronchoscopic aspiration (EUS‐B‐FNA) allowed for minimally invasive and simultaneous diagnosis and evaluation of the degree of invasion by echocardiography. EUS‐B‐FNA may be useful for the evaluation and diagnosis of tumours with cardiac invasion.

## Graphical abstract

EUS‐B‐FNA may be used to evaluate and diagnose tumours with cardiac invasion. Safe, minimally invasive procedures were sufficient to confirm the diagnosis in our patient and assess the degree of invasion, which also allowed for chemotherapy to be initiated promptly.

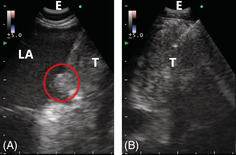

## CLINICAL IMAGE

**FIGURE 1:**
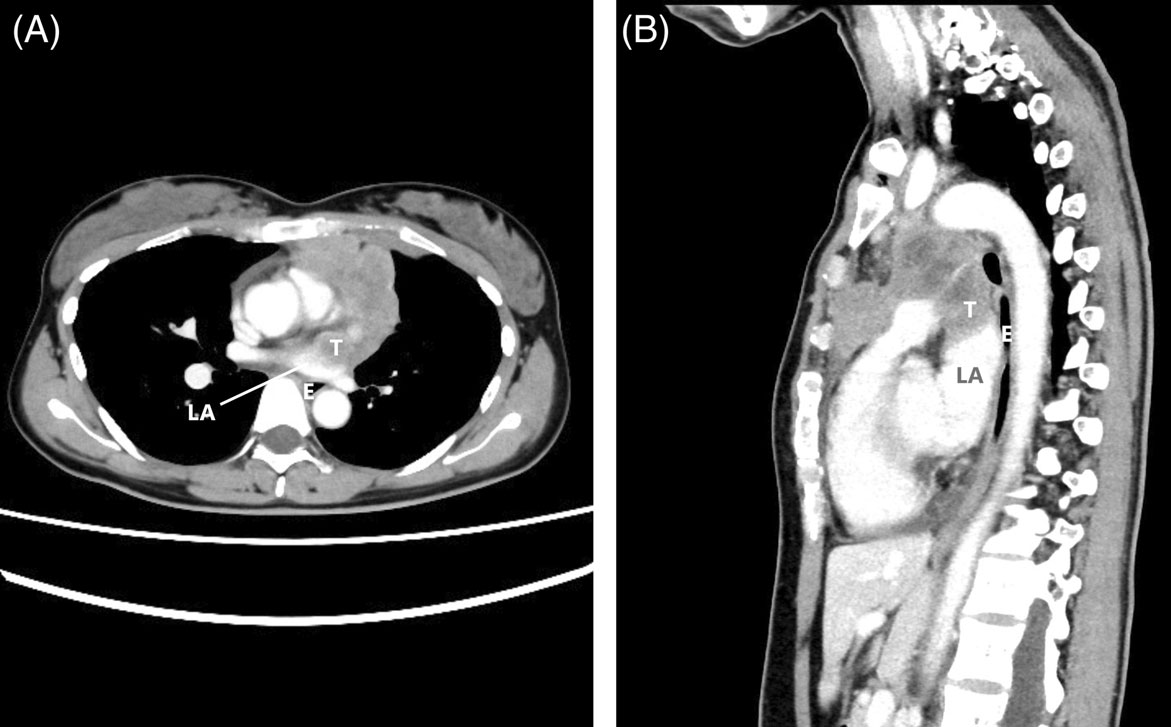
Enhanced computed tomography findings. Chest CT reveals suspected partial invasion of tumour adjacent to the heart into the left atrium. CT, computed tomography; LA, left atrium; T, tumour; E, oesophagus

**FIGURE 2:**
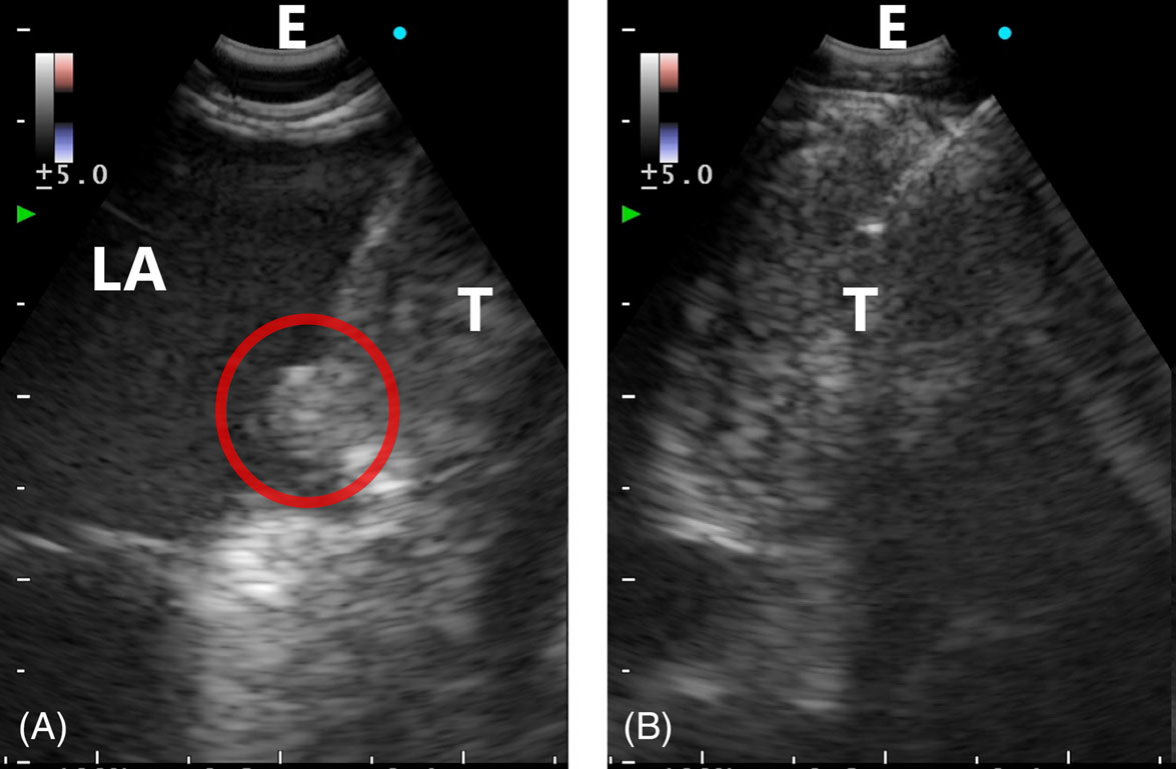
EUS‐B‐FNA findings. EUS‐B‐FNA findings show tumour invasion into the left atrium (A) and fine‐needle aspiration (B) of the mediastinal tumour adjacent to the heart. The red circle indicates tumour invasion. EUS‐B‐FNA, transesophageal ultrasound‐guided bronchoscopic aspiration

**FIGURE 3:**
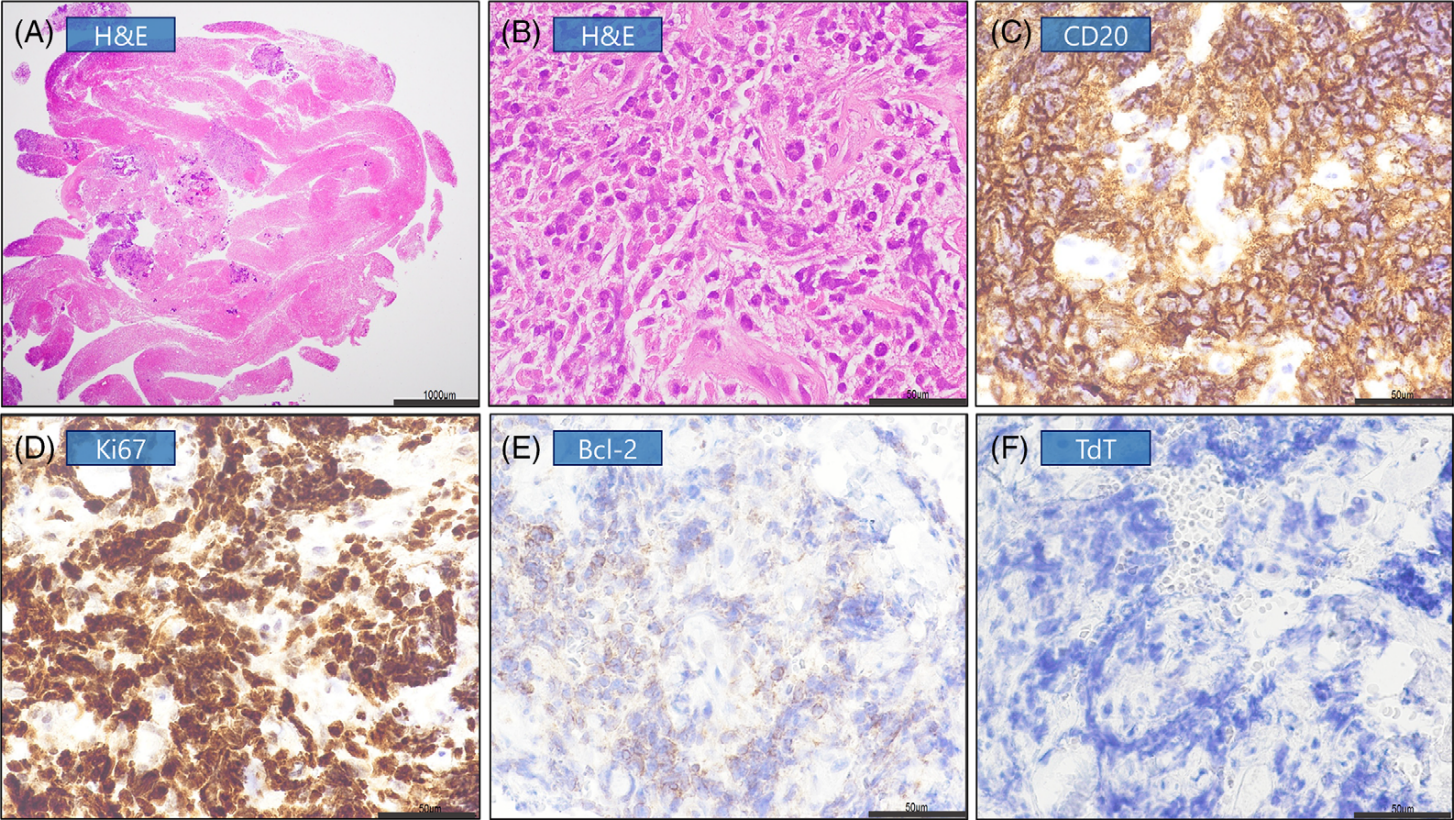
Pathological examination of specimens. H&E staining of specimens and immunohistochemistry for CD20, Ki67, Bcl‐2 and TdT. The tumour cells expressed CD20 and Ki67 (index 90%–100%) but not Bcl‐2 and TdT. The scale bar represents 1000 μm (A), 50 μm (B–F). H&E, haematoxylin and eosin

A 34‐year‐old woman presented with dyspnoea and a 1‐month history of coughing. Chest computed tomography showed a mass in the anterior mediastinum with suspected partial cardiac invasion (Figure 1). Transesophageal ultrasound‐guided bronchoscopic aspiration (EUS‐B‐FNA) confirmed tumour invasion of the left atrium (Video 1). Transesophageal needle biopsy of the tumour adjacent to the heart was performed while simultaneously visualizing the cardiac invasion (Figure 2). Diffuse large B‐cell lymphoma (DLBCL) diagnosis was confirmed through immunohistochemical staining (Figure 3). No arrhythmia, bleeding, extravasation, pericardial tamponade or other adverse events occurred. The patient received rituximab, etoposide, prednisolone, vincristine, cyclophosphamide and doxorubicin (R‐EPOCH), and the tumour shrank enough without developing tumour embolism. EUS‐B‐FNA enables safe real‐time sampling of lung tumours and mediastinal lymph nodes adjacent to the oesophagus.^1^ While a percutaneous needle biopsy could have been performed to confirm left atrial invasion, EUS‐B‐FNA allowed for minimally invasive and simultaneous diagnosis and evaluation of the degree of invasion by echocardiography, which resulted in prompt initiation of chemotherapy. Thus, EUS‐B‐FNA may be useful for evaluating and diagnosing tumours with cardiac invasion.^2^

**VIDEO 1:** EUS‐B‐FNA findings. Tumours invading the endocardium of the left atrium can be observed visible and hidden with the heartbeat.

## AUTHOR CONTRIBUTION

*Conceptualization*: Toshiyuki Sumi. *Data curation*: Yusuke Tanaka, Haruhiko Michimata and Daiki Nagayama. *Formal analysis*: Yoshiko Keira and Hiroki Watanabe. *Investigation*: Yoshiko Keira and Yuichi Yamada. *Roles/Writing – original draft*: Toshiyuki Sumi. *Writing* – *review & editing*: Hirofumi Chiba.

## CONFLICT OF INTEREST

None declared.

## ETHICS STATEMENT

The authors declare that appropriate written informed consent was obtained for the publication of this manuscript and accompanying images.

## Data Availability

Data sharing not applicable to this article as no datasets were generated or analysed during the current study.
